# Metallurgical Coke Production with Biomass Additives: Study of Biocoke Properties for Blast Furnace and Submerged Arc Furnace Purposes

**DOI:** 10.3390/ma15031147

**Published:** 2022-02-01

**Authors:** Oleg Bazaluk, Lina Kieush, Andrii Koveria, Johannes Schenk, Andreas Pfeiffer, Heng Zheng, Vasyl Lozynskyi

**Affiliations:** 1 Belt and Road Initiative Institute for Chinese-European Studies (BRIICES), Guangdong University of Petrochemical Technology, Maoming 525000, China; bazaluk@ukr.net; 2 Ferrous Metallurgy Chair, Montanuniversität Leoben, A-8700 Leoben, Austria; johannes.schenk@unileoben.ac.at (J.S.); andreas.pfeiffer@unileoben.ac.at (A.P.); heng.zheng@stud.unileoben.ac.at (H.Z.); 3 National Metallurgical Academy of Ukraine, 49600 Dnipro, Ukraine; 4 Department of Chemistry, Dnipro University of Technology, 49005 Dnipro, Ukraine; 5 Department of Mining Engineering and Education, Dnipro University of Technology, 49005 Dnipro, Ukraine; lvg.nmu@gmail.com

**Keywords:** biomass pellets, biocoke, coke, coke reactivity index, electrical resistivity, ferroalloys

## Abstract

Biocoke has the potential to reduce the fossil-based materials in metallurgical processes, along with mitigating anthropogenic CO_2_- and greenhouse gas (GHG) emissions. Reducing those emissions is possible by using bio-based carbon, which is CO_2_-neutral, as a partial replacement of fossil carbon. In this paper, the effect of adding 5, 10, 15, 30, and 45 wt.% biomass pellets on the reactivity, the physicomechanical, and electrical properties of biocoke was established to assess the possibility of using it as a fuel and reducing agent for a blast furnace (BF) or as a carbon source in a submerged arc furnace (SAF). Biocoke was obtained under laboratory conditions at final coking temperatures of 950 or 1100 °C. Research results indicate that for BF purposes, 5 wt.% biomass additives are the maximum as the reactivity increases and the strength after reaction with CO_2_ decreases. On the other hand, biocoke’s physicomechanical and electrical properties, obtained at a carbonization temperature of 950 °C, can be considered a promising option for the SAF.

## 1. Introduction

The metallurgical industry is one of the most energy-intensive industrial sectors and is responsible for significant anthropogenic GHG and CO_2_ emissions [1,2,3]. The main iron and steel production routes are BF/basic oxygen furnace (BOF), electric arc furnace (EAF), and SAF. Each process requires a heat and carbon source for carbothermal reduction. Using fossil-based materials as energy and carbon sources may have technological advantages [4,5]. However, achieving global goals to reduce anthropogenic GHG emissions is impossible without using alternative sources [6,7]. Approximately 5–10% of anthropogenic CO_2_ emissions are from metallurgical production. Most of these emissions come from smelting furnaces such as BF, EAF, and SAF [8]. This is due to their reliance on metallurgical coke [9,10,11]. However, biomass application can reduce emissions up to 12% in EAF or 58% in integrated steel production routes [12].

Bio-based fuel and reductants can replace the reductants’ fossil-based materials, reduce fossil carbon consumption, and mitigate harmful emissions in such processes as BF and SAF. This is because bio-based carbon can be classified as CO_2_-neutral, and therefore, replacing some of the fossil carbon, for instance, can result in lower CO_2_ emissions [12]. In BF processes, bio-based materials can be charged via tuyere injection, as lump-biocoke, and as a carbon source at the iron ore sinter plant [13]. In EAF, bio-based materials can perform the function of a conventional carbon source, namely, carburize the steel or create foaming slag to improve the energy efficiency of the melting process [14]. In the SAF process, bio-based materials can be used as bio-reductants in the production of ferroalloys [15]. The possibility of using alternative reducing agents in the production of ferroalloys is becoming more and more relevant and is presented in references [10,15,16,17].

Applying bio-based materials in metallurgical processes greatly impacts the total costs, energy consumption, operation stability, and, ultimately, final product quality. It is well known in the literature that biomass can be used in cokemaking [18,19,20,21,22], iron ore sintering [23,24,25,26,27], iron ore pellets production [28,29], and ironmaking [19,20,21,22,30,31,32,33,34,35,36,37,38].

The use of biomass in cokemaking is the most extensive issue since coke consumers are both BF and non-BF production. However, the conventional production of coke aims to ensure the quality corresponding to the BF process. In contrast, other consumers (except foundry production) require coke with higher reactivity, finer, and good mechanical strength but with more acceptable indicators of ash.

The coke reactivity index (CRI), coke strength after reaction with CO_2_ (CSR) [39], particles size, mechanical strength, ash, and sulfur content are the most important parameters that characterize the quality of the resulting coke and affect the BF performance.

Several papers show that the biocoke reactivity index increases, and biocoke strength after reaction with CO_2_ decreases when charcoal is added compared to coke [39,40]. However, El-Tawil et al. [41] produced coke at laboratory and technical scales from coking coal blends with 5% addition of high-temperature torrefied pelletized sawdust or 5% torrefied sawdust. In this study, the authors concluded that biocokes had similar thermogravimetric (TGA) reactivity trends compared to conventional coke. The attention to the study of the biocoke mechanical strength and the effect of biomass content on it has been studied in many papers [42,43,44]. It was found that the mechanical strength of coke (DI 150/15) carried out according to [45] decreases with an increase in the addition of biomass. The mechanical strength decrease is a consequence of the nature of the charcoal as a lump of non-coking coal [44]. Nevertheless, in a study by Silva et al. [46], it was shown that the addition of a calcined rice husk of 6% increased the average size of the coke without reducing its mechanical resistance. It also showed an increase in the DI 150/15 value, and the CRI values were close to the conventional coke.

Additionally, the influence of the charcoal size on the quality of the resulting biocoke was studied in [40,47], with a focus on strength as the quality parameter. In this connection, MacPhee et al. [40] noticed that biocoke produced using fine charcoal additive (-60 mesh) had much lower strength than coke made from a reference blend. However, the fractions of coarse charcoal (−3/8 + 1/4 in) allow higher quality biocoke.

In previous authors’ papers [19,48], the effect of adding 5 wt.% of biomass pellets on biocoke’s reactivity and microstructural properties has been studied. It was concluded that biomass pellets have a local impact on the coke structure. The basic requirements for fuels and reducing agents that interest various consumers were additionally analyzed. It was also concluded that a decrease in the effect of biomass on fuel quality could be achieved by either preliminary pyrolysis of biomass or using it in a pellet form.

For the SAF process, the main indicator of the quality of carbon-reductant is electrical resistivity, as an integral indicator of porosity, strength, hardness, and reactivity [15]. In addition, essential parameters of the reducing agent are the size and values of indicators of proximate analysis.

Using biomass instead of fossil fuels in metallurgy requires an integrated scientific approach. Thus, the current study attempted to consider the physicochemical and physicomechanical properties of biocokes obtained with different amounts of wood pellets in comparison with conventional coke obtained under the same conditions and under temperature conditions corresponding to industrial coking at 1100 °C, as well as under conditions when coke production will be directed to non-BF consumers and the final temperature of coke obtaining can be reduced [49] to 950 °C. Moreover, the effect on the biocoke properties of uniformly distributed (biomass particles) and unevenly distributed (biomass pellets) biomass within the coke matrix has been addressed. The quality of biocoke was compared with conventional coke and evaluated using indicators characterizing the properties of the lump carbon material in terms of reactivity, strength after reaction with CO_2_, ‘cold’ mechanical strength, and structural ordering macroscopic observations. The properties of the crack-free carbon material were also evaluated using structural strength, abrasive hardness, electrical resistance, and porosity. All the indicators mentioned above were used to characterize the properties of biocoke for various consumers in the metallurgical industry.

## 2. Materials and Methods

### 2.1. Sample Preparation

Coke was obtained from hard coals, widely used in the cokemaking production of the Dnipro Metallurgical Plant, Dnipro, Ukraine. The characteristics of the coking coals and the composition of the blend are shown in Table 1. Ukrainian industrial wood pellets were used as a biomass additive since woody biomass is considered a high-quality fuel [50] and the most suitable raw bio-material for metallurgical processes. Biomass particles were obtained by grinding biomass pellets to a particle size of less than 3 mm. Biomass characteristics are shown in Table 1. Petrographic analysis of coals and coal blend is presented in Table 2. The coal blend was prepared from Vitrinite-rich coals whose values vary in the range of 80–91%, and the random Vitrinite reflectance varies within 0.75–1.53%. The coal blend is characterized by a random Vitrinite reflectance value of 1.05%. It was reported [51] that the higher the Vitrinite reflectance of coal blend, the better the strength properties of the resultant coke. To achieve good coke strength, the Vitrinite reflectance should be within the range of 1.2–1.3% [52,53].

**Figure 1:**
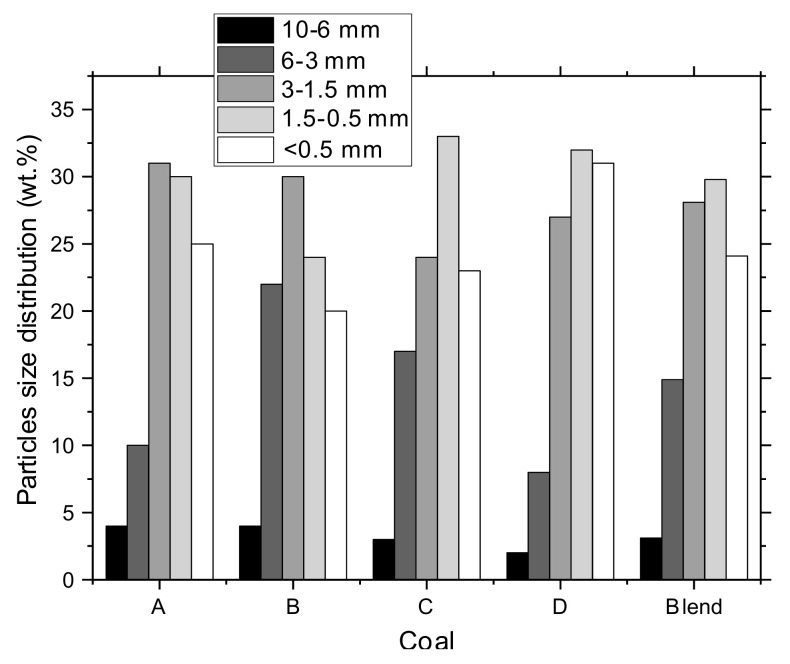
Particle size distribution of the coals and the coal blend.

A constant particle size distribution of coals was maintained for all blends to minimize its effect on the coke quality, as the study was focused on the influence of the additive. Figure 1 shows the coal particles size distribution within blends, wt.%. The distribution corresponds to 82% of fineness with the size <3 mm for the reference blend, while the fines content with the size <0.5 mm was 24%.

The diameter of the wood pellets was 8 mm, and the length varied from 4 to 22 mm. The biomass particle size distribution was close to the standard grinding of the coal blend and was 82% less than 3 mm. The choice of the biomass particle size distribution was due to the identity of the main size of the blend and to prevent an additional decrease in caking capacity [44].

The amount of wood pellet additives within the coal blends was 5, 10, 15, 30, or 45 wt.%. The decrease in the coal part of the blend by biomass pellets occurred proportionally for each type of coals, considering the stability of the particle size distribution of the coals within the blend. Additionally, the influence of adding 5 wt.% wood particles was studied. Biomass particles in 5 wt.% were added to the coal blend to compare the impact on the biocoke quality of homogeneously distributed particles with the unevenly distributed pellets. The carbonization of the blends was carried out under laboratory conditions at final temperatures of 950 °C and 1100 °C. The conditions for carrying out carbonization were presented in detail in [48]. Carbonization was carried out in an electric shaft-type laboratory furnace. The blend with a weight of 2 kg was loaded in a cylindrical retort. Carbonization was conducted 3 times at a temperature of 950 °C and 3 times at 1100 °C. The total carbonization time for 950 °C was 90 min, and 1100 °C was used for 105 min for each test.

### 2.2. Characterization

#### 2.2.1. Analysis of Yield and Particle Size Distribution

The obtained cokes/biocokes after naturally cooling to room temperature were subjected to the analysis of the cokes/biocokes yield (Yield, %). The cokes/biocokes yield was calculated using Equation (1).

(1) $$Yield=\frac{B}{A}\times 100\%$$

where *A* is the initial mass of the blend, g; *B* is the mass of coke/biocoke after carbonization, g.

Further, the resulting cokes/biocokes were sieved into sizes >25, 25–10, <10 mm according to ASTM D293/D293M-18 [54].

#### 2.2.2. Petrographic Analysis of Hard Coal

Preparation of coal samples, coal maceral composition determination, and Vitrinite reflectance determination were carried out in accordance with [55,56,57]. Random reflectance was measured on Vitrinite or altered Vitrinite using Lucia petrographic complex. The LECO PR-32 automatic press(LECO Instruments, Mumbai, India), the LECO GPX-300 grinding(LECO Instruments, Mumbai, India), and the polishing machine were used to prepare blocks. Reflectance was measured on polished surfaces under oil immersion with Refractive Index 1.515 using an Olympus research microscope (Labsol Enterprises, Gurugram, India) at a magnification of 50×, and Lucia Vitrinite 7.13 software was used. Measurements of the Vitrinite reflectance were performed at a mean of 200 points for each sample. Maceral analysis was based on the measurements of at least 500 points.

#### 2.2.3. Analysis of BJH (Barrett–Joyner–Halenda) Cumulative Pore Volume

Each sample weighing 1.0 g was taken into special test tubes and degassed for 3 h in a nitrogen atmosphere using a Flowprep 060 degasser (Micromeritics, Norcross, GA, USA). A temperature of 300 °C was chosen since the coke should not undergo interactions at this temperature. After degassing using the Tristar II 3020 instrument(Micromeritics, Norcross, GA, USA), the BJH cumulative pore volume of cokes and biocokes was studied. The measurements were carried out in a nitrogen atmosphere, according to [58]. The particle size for both coke and biocoke samples was set to 250 microns. Each test was carried out 3 times.

#### 2.2.4. Reactivity Index (CRI) and Strength after Reaction (CSR)

The determination of CO_2_ reactivity index and coke or biocoke strength after reaction with CO_2_ was carried out according to ISO 18894:2018 [59] using a vertical reduction furnace and a tumbler (Model TB 5000 of R.B. Automazione, Genoa, Italy). According to ISO 18894:2018, the values for CRI and CSR are calculated using Equations (2) and (3).

(2) $$CRI=100\times \frac{m_{0}-m_{1}}{x},$$

where $m_{0}$ is sample weight before reaction, g; $m_{1}$ is sample weight after reaction with CO_2_, g.

(3) $$CSR=100\times \frac{m_{2}}{m_{1}},$$

where $m_{2}$ is sample weight after reaction with CO_2_, g; $m_{1}$ is weight of coke greater than 10.0 mm after the tumbling, g.

#### 2.2.5. ‘Cold’ Mechanical Strength

The ‘cold’ mechanical strength of cokes and biocokes was measured in the Roga test tumbler according to [60,61]. Coke or biocoke samples weighing about 20 g (with the size of 19.0–22.4 mm as for CRI test) were placed in the tumbler drum, which contained seven iron ore sinter lumps, each weighing 25 g and 20 mm in size. The tumbler drum turned at a constant speed of 53 rpm and stopped after each 5 min period. The total time of tumbling was 25 min. After each rotation period, the material was removed from the tumbler drum and screened. The +3.15 mm material remaining in the drum after tumbling was weighed.

The mass of the +3.15 mm particles obtained after each rotation period was used to calculate the ’breakage index’ (BI) following Equation (4):

(4) $$BI=\frac{\frac{m_{5}+m_{25}}{2}+m_{10}+m_{15}+m_{20}}{4m_{i}}\times 100$$

where *BI* is the breakage index; *m_5_*, *m_10_*, *m_15_*, *m_20_, m_25_* are the mass of the +3.15 mm particles after each rotation period; *m_i_* is the initial mass of the coke or biocoke samples.

#### 2.2.6. Structural Strength

To determine the structural strength, coke and biocoke samples of 6–3 mm were prepared and filled into two special steel cylinders according to the GOST-9521 [62]. The volume of one sample was 50 cm^3^. The inner diameters of the cylinders were 25 ± 1 mm with a working part length of 310 ± 0.5 mm with steel plugs that were screwed on the end of the cylinders. Five steel balls with a diameter of 15.08 mm were also placed in the cylinders. These two cylinders were set through screws in the ’cross to cross’ position in brackets, which were put on a shaft rotating with 0.417 s^−1^ (25 rpm). During the test runs, the coke or biocoke cylinders performed 1000 revolutions, after which the contents of each of the cylinders were poured separately on a sieve with a mesh of 3 mm and 1 mm. Cokes or biocokes were sieved for 2 min to separate cokes or biocokes into 3–1 mm and 1–0 mm fractions. The cokes or biocokes yield of more than 1 mm from the initial weight in percent characterizes the structural strength of coke or biocoke.

#### 2.2.7. Abrasive Hardness

The abrasive hardness of coke or biocoke was determined by abrasion of an aluminum plate against the coke or biocoke powder and evaluating the loss of its mass. A total of 4–5 g dry coke or biocoke with a size of less than 0.5 mm was filled on an aluminum plate. A stamp was placed on top of the coke or biocoke samples, which was loaded to provide a plate pressure of 0.25 MPa. During the rotation of the rotor (500 rev.), the coke or biocoke samples abraded the aluminum plate. The weight loss of the aluminum plate for the test run (in milligrams) was taken as the value of abrasive strength. Five tests were performed for each coke or biocoke sample. In case of result deviation by more than 10%, it was not considered. A new aluminum plate was used for each test.

#### 2.2.8. Electrical Resistivity

The electrical resistivity measurement was carried out according to ISO 10143:2019 [63]. The method aims to determine the electrical resistivity of the bed of particles with a size of <2 mm obtained after the grinding and screening, located in the cylinder between two stainless steel plungers under a pressure of 3 MPa. The resistivity measured using a four-point mode is advantageous because it allows measuring resistivity close to the actual resistivity of the sample [64]. In addition, the four-point mode is more sensitive to detecting the difference between the two samples.

#### 2.2.9. X-ray Diffraction (XRD) Analysis

XRD analysis was conducted using a Bruker AXS D8 advance diffractometer with a lynxeye detector and a Cu x-ray tube. The wavelength of incident X-ray for Cu Kα radiation in this study was 1.5406 Å. XRD patterns were taken at long accumulation times (2 s). The X-ray intensity was measured in the range of 5° ≤ 2θ ≤ 90° with a scan speed of 2°·min^−1^. The d_002_ and crystallite height (L_c_) values were calculated from the 002-diffraction peak according to the Bragg law and Scherrer equation [65]. The crystallite width (L_a_) value was calculated from the 110-diffraction peak and the Scherrer equation.

#### 2.2.10. Microscopic Analysis

For slides preparation, the precision wet abrasive cut-off machine ATM Brillant 221 (QATM) and Opal 450 Embedding Machine (Spectrographic Ltd, Leeds, UK) were used with polishing steps of 9 µm and 3 µm. Keyence VHX digital microscope (Keyence, Itasca, IL, USA) was used for observing the macrostructure features of biocoke and biomass pellets.

## 3. Results and Discussion

### 3.1. Yield, Particle Size Distribution, and Proximate Analysis of Cokes and Biocokes

Table 3 shows the yield of coke and biocokes with different amounts of biomass addition at carbonization temperatures of 950 or 1100 °C, particle size distribution, and proximate analysis carried out according to [66]. The data of A^d^, VM^d^, and S_t_^d^ for cokes at 950 and 1100 °C and biocokes with 5 wt.% pellets at 950 and 1100 °C were noted in [48].

Compared with conventional coke, for which the yield was 74.5% at 950 °C, and 73.3% at 1100 °C, the yield of biocoke decreases with an increasing amount of biomass pellets for both carbonization temperatures, which is associated with a high content of volatiles in the initial biomass. Additionally, the yield of coke and biocoke decreased with an increase in the final carbonization temperature due to the deeper processes of destruction of the blend and the total release of volatiles. With an increase in the carbonization temperature, higher-molecular compounds undergo thermal destruction, and during the destruction of which volatile matters are released, and, consequently, the yield of coke decreases. This is evidenced by a decrease in VM^d^ for cokes obtained at 1100 °C. The ash content of coke after carbonization is consistently higher than that of the initial charge [67]; however, less ash biomass in the blend contributes to a decrease in the ash content of biocokes compared with the reference.

The use of biomass particles in comparison with pellets also has a noticeable effect on the size composition of the resulting biocoke. The size of cokes >25 mm, which is used for BF purposes, decreases with an increase in the percentage of biomass pellets due to increased fracturing and porosity [68]. Consequently, the amount of biocoke with 25–10 mm and <10 mm for non-BF purposes increases. It should be noted that the biomass particles, which, unlike pellets, are more evenly distributed over the blend, contributed to the greater breaking of the material, and the yield of >25 mm is lower compared to pellets. Moreover, for biocokes obtained at 1100 °C, the yield of >25 mm is greater than for 950 °C, the yield of <10 mm is less. This is due to increased carbonization temperature and cokes’ improved structure and mechanical strength.

### 3.2. Biocoke Characterization for Blast Furnace Purpose

Since it was previously found that with an increase in biomass addition, the reactivity of coke increases [30], this section considers the effect of 5 wt.% addition of biomass pellets as the optimal option for the possible use of biocoke in the BF.

**Figure 2:**
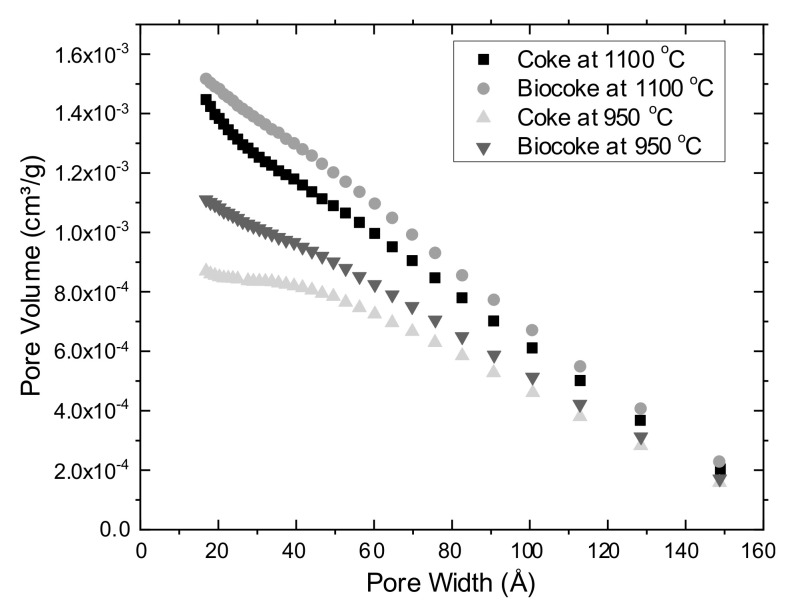
Cumulative pore volume for coke and biocoke (5 wt.% addition of biomass pellets) samples.

A variation of cumulative pore volume with pore width for coke and biocoke samples is given in Figure 2. The total cumulative volume of all coke and biocoke samples has different values. In all the cases, the maximum cumulative volume percentages of pores are in the ranges of 20–40 Å. It should be noted that, as for biocokes obtained at 950 °C and 1100 °C, the cumulative pore volume values are higher than for cokes at the same temperatures. This is due to charcoal pellets (here and after, which are applicable for carbon material after carbonization) increasing the cumulative pore volume.

**Figure 3:**
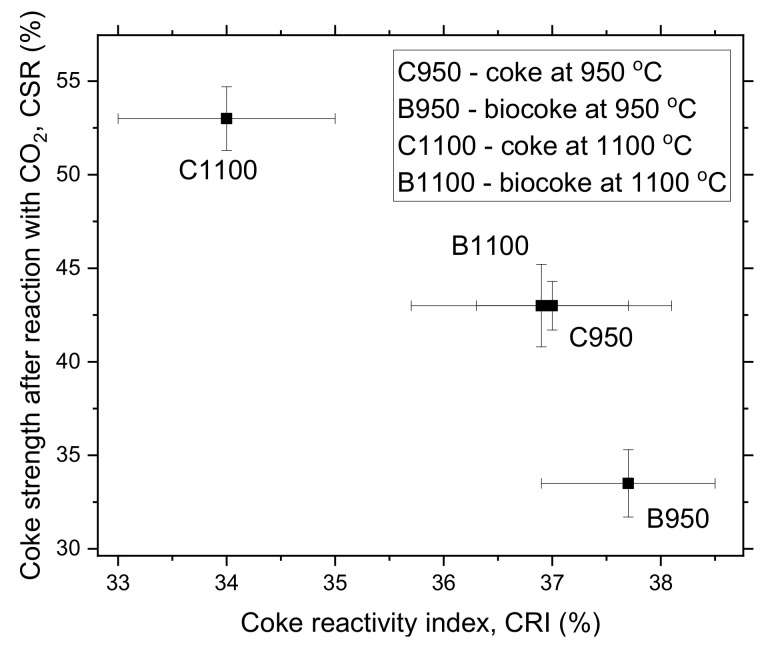
Relationship between coke reactivity index (CRI) and coke strength after reaction with CO_2_ (CSR) for samples of coke and biocoke with 5 wt.% biomass pellets; data from [48].

Figure 3 shows a clear line relationship between coke reactivity index and coke strength after reaction with CO_2_. This CRI and CSR parameters behavior is well known and was previously presented in [69]. According to Figure 3, the lower the reactivity index, the better the strength after reaction with CO_2_. In the case of biocoke, charcoal pellets reduce the proportion of anisotropic texture in the coke and increase its reactivity with CO_2_. Charcoal particles are isotropic [70] and have a poorer crystalline organization than anisotropic [71]. These isotropic textures react predominantly with CO_2_ [70,72] and contribute to the weakening of coke due to the formation of additional pores and causing a decrease in strength. However, CRI values for C950 and B950 are within the standard deviation. They show that adding 5 wt.% biomass pellets does not significantly change the quality, confirming the possibility of using biocoke instead of conventional coke.

**Figure 4:**
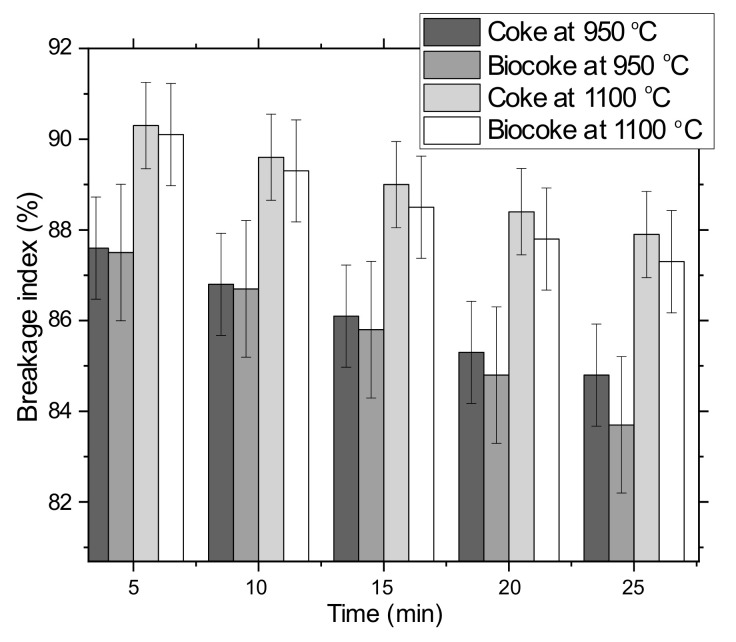
Breakage index of cokes and biocokes with 5 wt.% additives of biomass pellets.

The ‘cold’ mechanical strength values for coke and biocoke at 950 or 1100 °C are shown in Figure 4. It can be seen that the values for coke and biocoke at 1100 °C are higher in comparison with coke and biocoke obtained at 950 °C.

**Figure 5:**
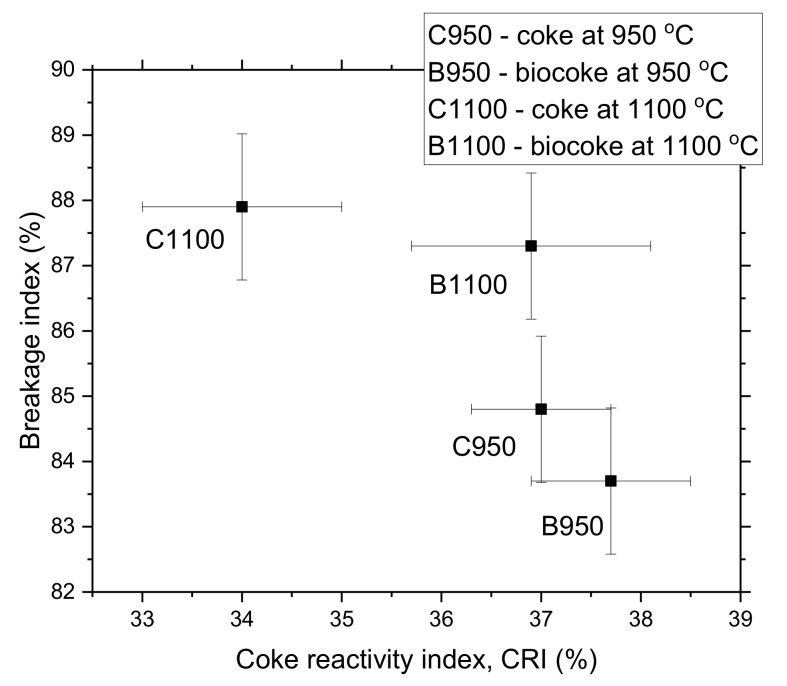
Relationship between coke reactivity index (CRI) and breakage index for coke and biocoke (5 wt.% biomass pellets) samples; selected data from [48].

The BI values for coke and biocoke at the same temperatures differ slightly and are within standard deviations. However, the presence of charcoal pellets reduces the mechanical properties of biocoke, which becomes more noticeable with an increase in the duration of the time of mechanical action on lumps of cokes or biocokes. Thus, with an increase in the total time of mechanical action, the difference in BI increased. The final ‘cold’ strength values follow the decreasing CSR. In addition, as shown in Figure 5, the CRI decreases with an increasing BI.

The BI for coke and biocoke samples at 1100 °C and the reference metallurgical coke in [60] have close values. It is important to note that the values of reactivity and strength are the course of the caking process of the coal blend upon heating. The caking process occurs on the surface of coal particles. Consequently, the presence of biomass pellets in the blend affects the formation of a porous coke matrix and its structure. Therefore, parameters of structural ordering in samples of initial biomass, charcoal pellets, coke, and biocoke at different carbonization temperatures were calculated and are presented in Table 4. The d_002_, L_c_, and L_a_ values for coke and biocoke at both carbonization temperatures were previously explained in [48]. To determine the parameters of the structural ordering of charcoal pellets, they were taken from biocokes obtained at temperatures of 950 and 1100 °C. This allowed comparing different carbon materials obtained under the same heat treatment conditions. The charcoal pellets within the coke matrix lead to a decrease in the ordering of the coke structure, an increase in the interplanar distance d_002_ between carbon hexagonal grids, and a decrease in L_a_. The presented values of L_c_ for coke are in the typical range according to [73].

**Figure 6:**
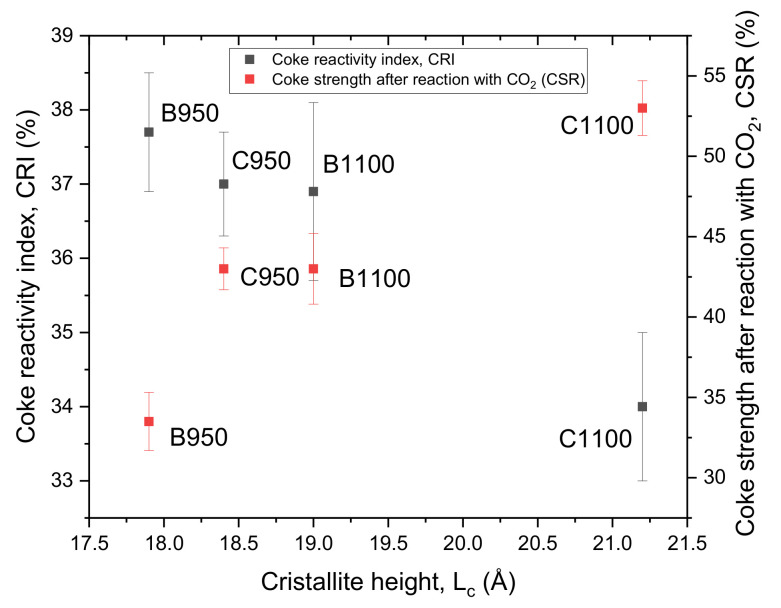
Relationship between coke reactivity index (CRI), coke strength after reaction with CO_2_ (CSR), and crystallite height (L_c_) for samples of cokes and biocokes with 5 wt.% biomass pellets; data from [48].

The relationship between CRI, CSR values, and crystallite height (L_c_) is shown in Figure 6. Reactivity decreases, and strength increases with increasing structure ordering due to improved two-dimensional orientation and an increase in the size of molecular-oriented domains [74]. According to the obtained values, the carbonization temperature of 1100 °C promotes the formation of crystallites with longitudinal dimensions (L_a_) larger than those in the transverse direction (L_c_), which causes coke anisotropy.

Compared to reference coke, charcoal pellets within the biocoke structure reduce its suitability for the BF process. The deterioration in properties will become more and more noticeable with an increasing amount of biomass and a decreasing final carbonization temperature. On the other hand, biomass may positively affect non-BF industries, such as SAF, by increasing the reactivity and reducing ash and sulfur for coke.

### 3.3. Biocoke Characterization for Submerged Arc Furnace Purpose

Table 5 presents the physicomechanical and electrical properties of biocoke and coke samples.

The structural strength of coke is the strength of its porous body, devoid of visible cracks. This indicator depends on the coal grains’ caking strength on the coke material and the thickness of the pore walls. Selected coke or biocokes samples obtained after carbonization at temperatures of 950 or 1100 °C have high values that meet the requirements for blast furnace coke >80% [75]. However, biomass pellets in 5 wt.% significantly reduce this indicator. At the same time, the decrease in the structural strength with the addition of biomass particles is even more significant. This can be explained by the different distribution of pellets and particles in the coke matrix and consequently by a large number of cracking centers, which are charcoal particles and, as a result, lead to greater destruction of coke.

The hardness of the crushed coke material is characterized by abrasive hardness. In addition to the strength of the lumps, coke and biocoke should have sufficient hardness, which is determined by the hardness of the pore walls, not weakened by cracks, and which is due to the degree of readiness and fusion of coal particles. Abrasive hardness has the highest values for coke samples, both at 950 °C and at 1100 °C. Further, with increasing biomass pellets, the abrasive hardness decreases.

Both indicators, namely, abrasive hardness and structural strength, have similar tendencies to deterioration with increasing biomass addition, since they reflect the quality of the carbon material at the structural level.

**Figure 7:**
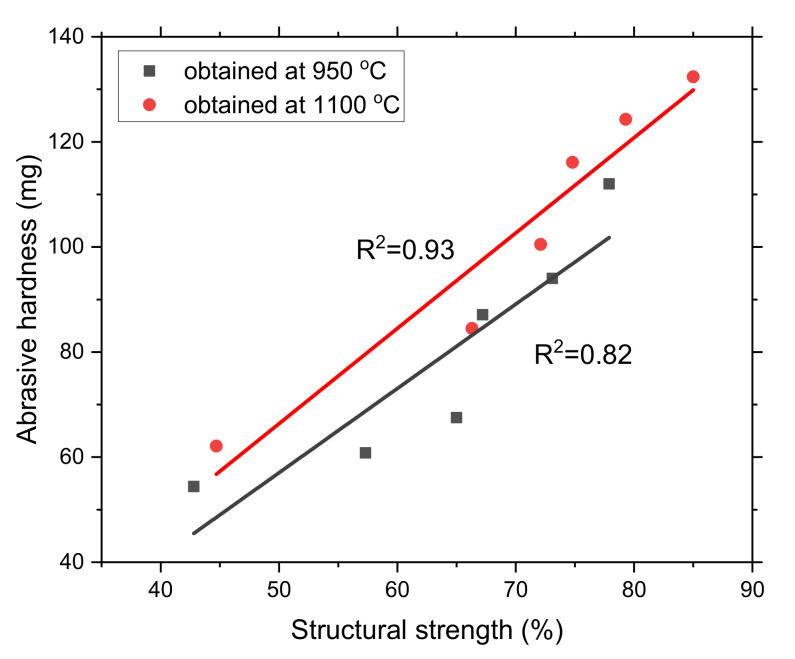
Relationship between structural strength and abrasive hardness for coke and biocoke carbonized at 950 °C and 1100 °C.

Figure 7 shows the relationship between structural strength and abrasive hardness for coke and biocoke at 950 °C and 1100 °C. As can be seen from Figure 7, the structural strength increases with an increase in the abrasive hardness of the material for both carbonization temperatures, and the dependence is closer with an increase in structural order, which occurs with an increase in the carbonization temperature.

The improvement of the mechanical properties of cokes and biocokes with an increase in the carbonization temperature can be explained by an increase in the amount of spatial arrangement of molecular-oriented domains, a decrease in the distance between carbon hexagonal grids-monolayers d_002_, and an increase in L_a_.

As shown from Table 5, the electrical resistivity of carbon-containing materials decreases with an increase in the carbonization temperature. It is in the range of 12.0–15.9 mΩm for 950 °C and 10.3–13.8 mΩm for 1100 °C. The electrical resistivity reflects the state of its carbon structure and the contact density of the residual material of coal grains and petrographic components inside the grains. Consequently, cokes obtained from poor-caking and petrographically heterogeneous and/or high-ash coals are characterized by a higher electrical resistivity. Thus, the electrical resistivity for coke at 950 °C and 1100 °C is lower in comparison to the values of biocoke. With an increasing amount of biomass pellets, the electrical resistivity increases. At the same time, the electrical resistivity decreased with a higher carbonization temperature, which is consistent with [15,16,76,77] and explained by removing oxygen-containing functional groups and the restructuring of the coke matrix. A decrease in electrical resistivity is also due to the formation and/or increased carbon crystallites, previously presented in [16,78]. Therefore, inclusions of charcoal with a different structure than coke increase the electrical resistivity of the biocoke samples.

**Figure 8:**
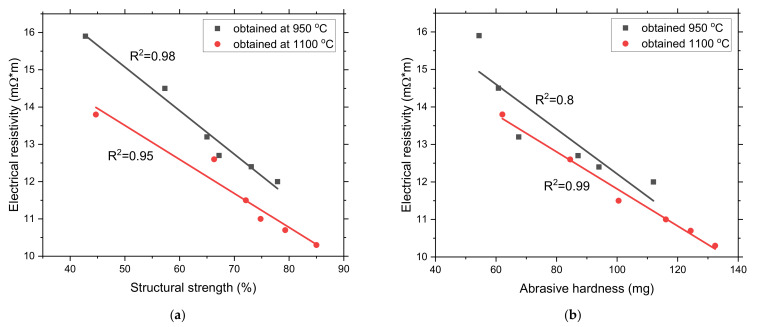
(**a**) Relationship between structural strength and electrical resistivity for cokes and biocokes carbonized at 950 °C and 1100 °C; (**b**) relationship between abrasive hardness and electrical resistivity for cokes and biocokes carbonized at 950 °C and 1100 °C.

As an essential indicator for SAF, electrical resistivity can be considered an integral characteristic of the coke properties, as evidenced by the linear dependences of the change in electrical resistance on the structural strength and abrasive hardness, which are presented in Figure 8a,b.

The electrical resistance decreases with the increased structural strength and abrasive hardness. It should also be noted that the value of electrical resistance varies in direct proportion to the reactivity [76]. With an increase in the electrical resistance values, the reactivity of coke will increase, which is important for many non-BF industries [79].

The requirements for the reactivity, mechanical strength, and electrical resistance of the carbon material may not be as stringent [80] as for BF, which allows considering the use of biocoke with increased participation of biomass for SAF.

**Figure 9:**
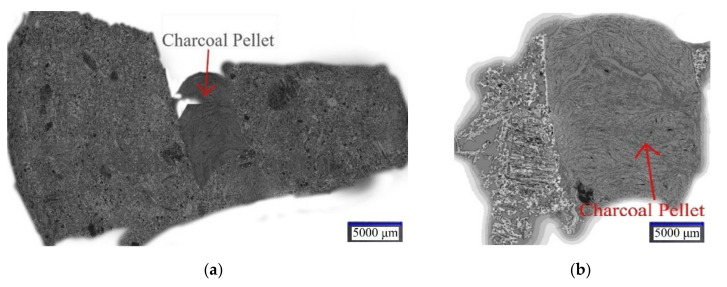
**(a)** Image of the macrostructure of the biocoke lump; magnification ×20; **(b)** image of charcoal pellets within the coke structure; magnification ×50.

Lumps of biocoke and charcoal pellets inside the coke structure were examined using a digital microscope. As Figure 9a,b shows, charcoal pellets have a clear boundary in the coke matrix, as evidenced by well-defined inclusion limits, and are thus the cause of cracking. In addition, given the high porosity of charcoal, their presence leads to a decrease in the strength of the biocoke.

Additionally, Figure 9a,b shows that in heating the blend, which consists of coals and biomass pellets, shrinkage processes occur for coals and biomass pellets separately, with the formation of the structure of coke and charcoal respectively. Thus, the resulting biocoke with the addition of biomass pellets can be a good substitute for the conventional reducing agent for SAF with the potential to reduce CO_2_ emissions.

## 4. Conclusions

This paper provided insight into the experimental study relating to the effect of biomass on coke quality for BF and SAF applications. A study of the influence of the high blending ratio of biomass pellets from 10–45 wt.% on the properties of biocoke has been carried out, and the choice of such biocoke as a bio-reducer for non-BF consumers, for example, SAF, has been considered. The comparison of biocoke’s physicomechanical properties differs in the biomass used to produce biocoke, namely, the comparison of biomass pellets and biomass particles (both for 5 wt.% ratios). The properties of biocoke, both lumpy and free from cracks, have been studied. The results of the physicomechanical properties of biocokes can be useful for many technological processes where fuel and carbon-reducing agents are used. From the obtained results, the following conclusions can be drawn:

− Maximum cumulative volume percentages of pores both for cokes and biocokes (5 wt.% addition of biomass pellets) are in the ranges of 20–40 Å. However, for biocoke samples at 950 °C and 1100 °C, the cumulative pore volume values are higher than cokes at the same temperatures. This is due to the presence of charcoal pellets, which increase the cumulative pore volume;
− A relationship between CRI and CSR parameters was observed for both conventional coke and biocoke (5 wt.% addition of biomass pellets) at both carbonization temperatures;
− ‘Cold’ mechanical strength values for coke and biocoke (5 wt.% addition of biomass pellets) at the same temperatures differ insignificantly. However, the presence of biomass pellets in biocoke reduces the strength properties of biocoke, which becomes more noticeable with an increase in the time of mechanical action on lumps of coke or biocoke. The presence of biomass pellets affects the formation of a porous coke matrix and cracks within the structure, resulting in a deterioration in coke strength;
− As for structural strength, the abrasive hardness increases with increasing carbonization temperature for coke and biocoke (5, 10, 15, 30, and 45 wt.%) samples. It was observed that both indicators have similar behavior;
− It was found that the electrical resistivity of carbon-containing materials decreases with an increase in the carbonization temperature, which is associated with the formation and/or increase of carbon crystallites and was in the range of 12.0–15.9 mΩm for 950 °C and 10.3–13.8 mΩm for 1100 °C. An increase in the values of electrical resistivity, as well as reactivity, indicates a decrease in the orderliness of the coke structure;
− Images of lumpy biocoke allow concluding that charcoal pellets have a clear boundary in the coke matrix as they are present in the form of inclusions, acting as cracking centers. The presence of charcoal pellets increases overall porosity and reactivity.

As can be seen from the results, the presence of charcoal pellets in the coke structure reduces its quality for the BF process. The deterioration of properties becomes more and more noticeable with an increase in the amount of biomass addition and a decrease in the final carbonization temperature. However, biocoke for non-BF consumers, particularly SAF, can be obtained with a high amount of biomass additions and at a reduced temperature of 950 °C, which will shorten the carbonization time and increase the productivity of biocoke production.

## Figures and Tables

**Table 1:** Blend component characteristics [48] (reprinted from Fuel, 309, Lina Kieush, Johannes Schenk, Andreas Pfeiffer, Andrii Koveria, Gerd Rantitsch, Horst Hopfinger, Investigation on the influence of wood pellets on the reactivity of coke with CO_2_ and its microstructure properties, 122151, copyright (2022), with permission from Elsevier (or applicable society copyright owner)).

Component	Participation within the Blend, wt.%	Proximate Analysis, wt.%				Ultimate Analysis, wt.%				Fixed Carbon **, wt.%
		M^a^	A^d^	VM^d^	S^d^_t_	C^d^	H^d^	N^d^	O^d*^	
Coal A	30	1.4	11.0	33.6	0.82	74.49	5.09	1.61	6.99	55.4
Coal B	25	1.5	11.0	30.3	0.68	75.38	4.77	1.54	6.63	58.7
Coal C	30	3.8	8.4	23.2	0.32	82.61	4.78	1.43	2.46	68.4
Coal D	15	1.2	8.9	16.7	0.50	80.32	4.15	1.38	4.75	74.4
Coal blend	100	2.1	9.9	27.1	0.58	78.02	4.78	1.50	5.22	63.0
Wood biomass	5–45	9.1	5.6	73.1	0.09	45.34	5.86	0.58	42.54	21.3

M^a^ is moisture (air-dried basis); A^d^ is ash (dry basis); VM^d^ is volatile matter (dry basis); S^d^_t_ is total sulfur (dry basis); C^d^ is carbon (dry basis); H^d^ is hydrogen (dry basis); N^d^ is nitrogen (dry basis); O^d^ is oxygen (dry basis). *Calculated by difference, O^d^, % = 100 − C ^d^ − H^d^ − N^d^ − S^d^ − A^d^. ** Calculated by equation, fixed carbon, % = 100 − (%V^d^ − %A^d^).

**Table 2:** Petrographic analysis of hard coals and coal blend.

Component	Participation within the blend, wt.%	Random Vitrinite reflectance (R_r_), %	Vitrinite (Vt), %	Inertenite (I), %	Liptinite (L), %
Coal A	30	0.75	80	17	2
Coal B	25	0.93	87	10	2
Coal C	30	1.22	91	8	0
Coal D	15	1.53	86	12	0
Blend	100	1.05	86	12	1.1

**Table 3:** Yield, particle size distribution, and proximate analysis of coke and biocoke with different amounts of additives.

Blends	Type of Additives	Additive, wt.%	Yield of Coke/Biocoke, %	Particle Size Distribution, %			Proximate Analysis, %			**Fixed Carbon, wt.%**
				>25 mm	25–10 mm	<10 mm	A^d^	VM^d^	S_t_^d^	
Obtained at 950 °C										
Coke			74.5	93.9	1.3	5.7	10.9	1.64	0.24	87.5
Biocoke	Pellets	5	70.9	92.7	2.5	4.8	10.3	1.55	0.23	88.2
		10	69.0	88.6	2.3	9.1	10.0	1.50	0.22	88.5
		15	66.5	79.3	4.9	15.8	9.7	2.70	0.21	87.6
		30	59.6	55.3	9.5	35.2	8.4	2.33	0.19	89.3
		45	52.5	30.8	12.7	56.5	6.0	1.85	0.16	92.2
	Particles	5	70.7	89.2	2.4	8.4	10.3	1.52	0.23	88.2
Obtained at 1100 °C										
Coke			73.3	93.6	0.9	5.5	11.1	1.46	0.23	87.4
Biocoke	Pellets	5	69.8	92.8	2.6	4.6	10.8	1.40	0.22	87.8
		10	68.7	88.9	3.4	7.7	10.5	1.42	0.2	88.1
		15	66.3	81.4	3.8	14.8	10.2	1.57	0.18	88.2
		30	59.3	60.1	7.9	32.0	8.9	1.53	0.17	89.6
		45	52.4	36.7	13.5	49.8	5.8	1.80	0.16	92.4
	Particles	5	69.4	90.5	3.8	5.7	10.8	1.50	0.22	87.7

**Table 4:** Parameters of structural ordering in biomass, charcoal pellets, coke, and biocoke samples at different carbonization temperatures; selected data from [48].

Material	Parameters of Structural Ordering			
	d_002_, Å	L_c_, Å	L_a_, Å	L_c_/L_a_
Coke at 950 °C	3.50	18.4	34.3	0.54
Biocoke with 5 wt.%, at 950 °C	3.52	17.9	32.7	0.55
Coke at 1100 °C	3.43	21.2	37.5	0.56
Biocoke with 5 wt.%, at 1100 °C	3.49	19.0	35.8	0.53
Charcoal pellets at 950 °C	3.70	16.9	28.3	0.60
Charcoal pellets at 1100 °C	3.61	17.6	29.8	0.59
Initial biomass	3.93	15.6	n/a	n/a

n/a—not available.

**Table 5:** Physicomechanical and electrical properties of biocoke and coke samples.

Blends	Type of Additives	Additive, wt.%	Structural Strength, %	Abrasive Hardness, mg	Electrical Resistivity, mΩ·m
Obtained at 950 °C					
Coke			77.9	112.0	12.0
Biocoke	Pellets	5	73.1	94.0	12.4
		10	67.2	87.1	12.7
		15	65.5	67.5	13.2
		30	57.3	60.8	14.5
		45	42.8	54.4	15.9
	Particles	5	69.7	103.0	12.4
Obtained at 1100 °C					
Coke			85.0	132.4	10.3
Biocoke	Pellets	5	79.3	124.3	10.7
		10	74.8	116.1	11.0
		15	72.1	100.5	11.5
		30	66.3	84.5	12.6
		45	44.7	62.1	13.8
	Particles	5	76.8	128.0	10.7

## Data Availability

Data are contained within the article.
